# Rheostats, not switches: Nuclear receptor dosage tunes somatic-to-iPSC reprogramming

**DOI:** 10.1016/j.stemcr.2026.102871

**Published:** 2026-04-02

**Authors:** Constantinos Chronis

**Affiliations:** 1 Department of Biochemistry and Molecular Genetics, University of Illinois Chicago, MBRB 2370, 900 S Ashland Av, Chicago, IL 60607, USA

## Abstract

Nuclear receptors are modular transcription factors regulated by ligands, chromatin context, and expression level. Wang et al. 2026, in this issue of Stem Cell Reports show that Rora controls OKS-mediated reprogramming in a dose-dependent manner: moderate expression enhances iPSC formation, while high levels inhibit it, linking quantitative Rora control to immune and WNT signaling during cell fate conversion.

## Main text

The conversion of somatic cells into induced pluripotent stem cells (iPSCs), using defined transcription factors (Takahashi and Yamanaka 2006) or chemical signaling approaches (Hou et al., 2013), is a landmark example of cellular plasticity. Although the core Yamanaka factors (Oct4,Sox2,Klf4,cMyc; OSKM) have been extensively studied (Soufi et al., 2012; Chronis et al., 2017), much less is known about how auxiliary regulators, particularly their quantitative expression, shape reprogramming trajectories. Developmental systems typically rely on graded signaling and gene dosage rather than simple on-off switches to generate distinct cellular states (Ashe and Briscoe 2006). In this issue, Wang et al. address this quantitative dimension by systematically screening 49 nuclear receptors during OKS-mediated reprogramming of mouse embryonic fibroblasts. Nuclear receptors, with their modular DNA-binding, ligand-binding, and regulatory N-terminal domains, are inherently sensitive to context- and expression levels, making them well suited for this analysis. This systematic approach identified the ROR subfamily as consistent enhancers of OCT4-GFP+ iPSC formation, positioning Rora as a quantitatively tuned regulator of reprogramming efficiency.

A central conceptual advance of this study is the discovery that Rora acts in a dose-dependent manner: low/moderate expression enhances iPSC colony formation, whereas higher levels inhibit it. Rather than producing a simple linear response, Rora generates distinct outcomes at different expression thresholds. This finding builds on prior work (Ho et al., 2013) demonstrating that reprogramming is highly sensitive to stage-specific signaling inputs. For example, although WNT signaling supports embryonic stem cell renewal, it inhibits early reprogramming but becomes required and stimulatory at late reprogramming stages. Wang et al. extend this paradigm by showing that not only the timing but also the magnitude of regulatory input influences reprogramming outcomes. Domain analyses further reveal that the DNA- and ligand-binding domains mediate Rora’s pro-reprogramming activity, whereas the N-terminal domain drives inhibition at high expression, providing a structural basis for this dose-dependent switch.

To clarify the mechanisms underlying Rora’s reprogramming effect, the authors performed RNA sequencing across varying Rora expression levels and found that moderate Rora expression suppresses interferon-γ (IFN-γ) signaling at both the transcript and protein levels. This is significant because prior studies (Guo et al., 2019) have established that innate immune activation and IFN-γ act as barriers to reprogramming, with IFN-γ known to impede the final transition to pluripotency. Consistent with this, exogenous IFN-γ treatment eliminates the Rora-mediated enhancement of iPSC formation, directly linking reduced IFN signaling to improved reprogramming efficiency. Together, these findings identify Rora as a quantitative modulator of a well-established inflammatory barrier.

In contrast, high Rora expression is associated with reduced WNT pathway activity, offering a likely explanation for the decreased colony formation observed at elevated Rora levels. Because WNT signaling plays stage-specific roles during reprogramming, its suppression in the context of excessive Rora expression can be consistent with impaired progression toward iPSCs if it acts at late stages of reprogramming. Activation of WNT signaling with the GSK3 inhibitor CHIR99021 restores colony formation under these conditions, directly linking reduced WNT output to the inhibitory phenotype. Together with the attenuation of IFN/ISG signaling at intermediate Rora levels, these results indicate that Rora dosage coordinately influences inflammatory and WNT pathways, defining a narrow quantitative window that supports efficient reprogramming.

Beyond transcriptomic analyses, Wang et al. integrate CUT&Tag, ATAC-seq, and immunoprecipitation-mass spectrometry to define how Rora dosage reshapes chromatin occupancy and regulatory networks. Condition-wide clustering of CUT&Tag peaks across Rora^Low^, Rora^High^, and domain mutants reveals distinct occupancy modules that align with transcriptional changes, including enrichment of WNT- and cytoskeleton-related programs. Most Rora binding occurs within pre-accessible chromatin early in reprogramming, suggesting it modulates activity at established regulatory regions rather than engaging a slurry of new sites. Notably, all Rora conditions are associated with fewer new genomic sites (measured by ATAC) opening overall, suggesting more selective or divergent engagement of OSKM at pluripotency-associated loci, whereas fibroblast-associated regions are consistently closed across conditions.

Proteomic analyses further show that the Rora^High^ population is enriched for mesenchymal and fibroblast-associated interaction modules, suggesting that excessive Rora expression reinforces aspects of the initial fibroblast program. Similar maintenance of mesenchymal identity has been implicated as a barrier to reprogramming for other signaling-responsive transcription factors, such as AP-1 (Liu et al., 2015; Chronis et al., 2017). Together, these findings suggest that high Rora levels may impede pluripotency acquisition by both dampening pro-pluripotency signaling and stabilizing lineage-specific networks. How these dosage-dependent chromatin and interaction changes intersect with OSKM binding dynamics remains an important open question.

The implications of this work extend beyond Rora itself. Although reprogramming studies have largely focused on transcription factor identity and OSKM stoichiometry (Carey et al., 2011), Wang et al. demonstrate that the expression level of a single auxiliary regulator can substantially alter cell fate outcomes. The tunable behavior of Rora illustrates a broader principle: transcription factors function as rheostats rather than simple switches, with expression levels encoding distinct regulatory states. In this context, nuclear receptor abundance emerges as a tunable variable that influences signaling balance, chromatin dynamics, and reprogramming barriers. Moreover, the modular structure of nuclear receptors suggests opportunities for rational design, where domain-specific modifications—such as targeting the N-terminal domain—could uncouple beneficial from inhibitory effects. Together, these findings underscore that transcription factor dosage, not just identity, is a key determinant of reprogramming efficiency and highlight nuclear receptor modulation as a strategic avenue for engineering cell plasticity.
